# Whole-genome analyses of human adenovirus type 55 emerged in Tibet, Sichuan and Yunnan in China, in 2016

**DOI:** 10.1371/journal.pone.0189625

**Published:** 2017-12-14

**Authors:** Wenbo Wang, Yuan Liu, Yifan Zhou, Liangqi Gu, Lin Zhang, Xuelian Zhang, Maomao Chen, Ziying Zou, Wei Qiu, Xiaobing Hu, Quanshui Fan

**Affiliations:** 1 Center for Disease Control and Prevention of Chengdu Military Region, Chengdu, China; 2 Department of Clinical Laboratory, Chengdu Military General Hospital, Chengdu, China; Johns Hopkins University Bloomberg School of Public Health, UNITED STATES

## Abstract

Three outbreaks of acute respiratory disease occurred at military camps in 2016 at Tibet, Sichuan and Yunnan province, China. The pathogen induced these three outbreaks were all confirmed as HAdV-55 by genotype-specific PCR. The outbreak in Tibet was the first report that HAdV-55 occurred in the high altitude (HA, above sea level 3658 m). This study aims to determine the gene variation and evolution characteristics of these viral strains. Three strains of adenoviruses, LS89/Tibet/2016 (GenBank accession no. KY002683), SF04/SC/2016 (GenBank accession no. KY002684) and KM03/YN/2016 (GenBank accession no. KY002685) were obtained and confirmed by wholegenome sequencing. No multi-gene fragments recombination were found in these isolated HAdV-55 virus compared with previous reported HAdV-55 strains in China. The outbreaks in Tibet and in Sichuan continuously occurred. Virus isolated from Tibet (LS89/Tibet/2016) and Sichuan (SF04/SC/2016) had a similar mutation pattern and had a closer genetic evolutionary distance than KM03/YN/2016 strain, which indicates that the pathogens causing these two outbreaks may be of the same origin. Moreover, we found that heating was an effective way to inactive these viruses, which provide valuable information for the development of HAdV-55 vaccines. Our data provide new information for genetic evolution of HAdV-55, and contribute to the prevention and control of HAdV-55 infection in the future.

## Introduction

Human adenoviruses (HAdV) is a non-enveloped, double-stranded linear DNA virus, with a diameter of 70~90 nm. HAdV usually causes acute respiratory diseases (ARD), which had become a leading cause of outbreaks in community, school and military camps [1,2]. To date, there are more than 69 HAdV genotypes which grouped into 7 species (A-G) based on the antigenic variants of the capsid protein [3].

Two serotypes from species B, HAdV-3 and HAdV-7, are the common pathogens causing acute respiratory infection outbreaks in China previously [4]. However, in recent years human adenovirus type 55 (HAdV-55, species B) associated ARD outbreaks increased gradually [5–10]. HAdV-55 is a re-emergent pathogen arised from recombination of hexon gene between HAdV-11 and HAdV-14 strain [5,6]. The first reported HAdV-55 associated ARD outbreak occurred in a senior high school in Shanxi Province of China in 2006, and the QS-DLL strain of HAdV-55 was isolated from this outbreak [5]. HAdV-55 infection causes both mild and severe diseases, presenting clinical signs and symptoms including high fever, cough, sore throat, bronchitis and pneumonia, and sometimes is life-threatening [7].

Three outbreaks of acute respiratory disease occurred at military camps in Tibet (January 2016 to March 2016), Sichuan (February 2016 to March 2016) and Yunnan (Jun 2016) respectively, in China. The outbreaks in Tibet and in Sichuan were continuously happened. Pathogens induced these three outbreaks were all confirmed as HAdV-55 by qPCR and sequencing. The outbreak in Tibet was the first report that HAdV-55 occurred in the high altitude (HA, above sea level 3658 m). To analyze the mutation features of these viral strains, HAdV-55 virus caused these three outbreaks were isolated from swabs. Three strains of HAdV-55 virus, LS89/Tibet/2016, SF04/SC/2016 and KM03/YN/2016, were obtained and wholegenome sequences were submitted to GenBank. Sequence analysis was performed between these HAdV-55 strains and previous reported HAdV-55 virus in China. The gene variation of these three strains of HAdV-55 virus were not significant and no multi-gene fragments recombination were found. Laboratory characteristics, including thermostability and ultraviolet inactivation of these virus were tested.

## Materials and methods

### Ethics statement

This study was approved by the Medical Ethics Committee of Center for Disease Control and Prevention of Chengdu Military Region. The patients’ identification information had been removed. This study aims to analysis the gene variation of HAdV-B55 virus isolated from swab samples and does not involve patients’ identification information. Therefore, the need for informed consent was waived by the Medical Ethics Committee.

### Cells

The human laryngeal cancer cell line HEp-2 (ATCC catalog CCL-23) was purchased from BioVector NTCC Inc, Beijing, China. and cultured under standard conditions in DMEM (Life Technologies, CA, USA) supplemented with 10% fetal bovine serum (Life Technologies) plus 100 μg/mL of penicillin-streptomycin (Life Technologies) at 37°C in the presence of 5% CO_2_.

### Virus isolation

Swab samples were collected from patients with febrile respiratory infectious illness. HEp-2 cells were cultured in 24-well plates at 70% density and incubated with 100 μL swab sample for 6 hours, followed by the addition of fresh maintenance medium (DMEM, 2% FBS). The culture plates were incubated at 37°C in 5% CO_2_. The cytopathic effect (CPE) of inoculated cells were observed every day. Cell cultrues were harvested when the morphology of the cells corresponding to mid to full CPE (approximately 80% CPE). The harvested cell cultures were pooled and pelleted by centrifugation at 1000g for 5 min, which is the 1^st^ generation virus stocks. The 2^nd^ generation virus stocks were produced with 1^st^ generation virus stocks infected Hep-2 cells, and so as the 3^rd^ generation virus stocks. The 3^rd^ generation virus stocks were used for whole-genome analyses and in vitro infection experiments.

A wild type (WT) of HAdV-55 strain Y16/SX/2011 was obtained from Beijing Institute of Microbiology and Epidemiology [11].

### Acquisition of whole genome sequences and phylogenetic analysis

Twelve PCR products covering the entire genome of HAdV-B55 were obtained. Whole genome sequences were obtained using sequencing. Primers used were shown in S1 Table. Alignment of sequences and phylogenetic analysis was performed using DNAMAN software (Lynnon BioSoft). Phylogenetic trees were constructed using MEGA version 6.06 by neighbor-joining method with 1000 bootstrap replicates. The genome sequences of 21 reference HAdV strains were from GenBank. All data and accession numbers of HAdV strains used in this study are listed in Additional file: S2 Table.

### Quantitation of virus titer and virus growth kinetics assay

The virus titers were enumerated using the Reed and Muench method [12]. Briefly, HEp-2 cells (1×10^4^ cells per well) were seeded overnight in 96-well plates. Frozen virus stocks were diluted (10^−1^) in sterile 1% aqueous sodium deoxycholate (Sigma Aldrich) and mixed for 15 min to disaggregate virus. The virus was then subjected to 10-fold serial dilution in maintenance medium (DMEM, 2% FBS) and 100 μL of the diluted virus from the 10^−3^ dilution onwards was added into each well of HEp-2 cells. Plates were incubated at 37°C and observed daily using an inverted phase contrast microscope for the appearance of distinct CPE. Virus titers were reported as 50% cell culture-infectious doses per volume (CCID_50_/mL).

HEp-2 cells in DMEM containing 2% FBS were infected with viruses at an 100 CCID_50_ and incubated at 37°C. Supernatants and cells were harvested at 1, 3, 5 and 7 d post-infection. The intracellular and extracellular viral levels were determined by real-time quantitative PCR (qPCR) assays.

### Cell viability assay

Cell viability was determined using an Cell Counting Kit (CCK-8, Transgen, Beijing, China) according to the manufacturer’s protocol. Briefly, 1.5×10^4^ cells were seeded into each well of a standard 96-well plate and incubated at 37°C with 5% CO_2_. After overnight culture, the media was removed, and the cells were infected with different virus strains. Infected and uninfected cells were assayed at 12, 24 and 48 h post-infection using CCK8 kit. The absorbance was determined at 490 nm using a microplate spectrophotometer (Bio-Rad, Hercules, CA, USA).

### Thermostability analysis

The viruses were incubated at 56°C for 30 minutes. HEp-2 cells were infected with normal and thermo-inactive viruses for 6 hours. Viruses were removed form the cells and fresh DMEM were added. Intracellular viral DNA levels were determined using real-time qPCR method at 24 hours post infection.

### Ultraviolet irradiation analysis

HAdV-55 virus were inactive by ultraviolet (UV) irradiation at a wavelength of 254 nm. Specifically, viruses were placed in a 48-well plate and formed thin films which are necessary to ensure complete penetration of the UV light. Viruses were treated with UV irradiation for 30 minutes. The control viruses were equally placed in a 48-well plates without ultraviolet irradiation. HEp-2 cells were infected with UV-inactive and control viruses. Viral DNA levels were determined using real-time qPCR method at 24 hours after infection.

### Viral DNA extraction and real-time qPCR

Cells were homogenized, and then viral DNA was extracted using a PureLink™ Viral RNA/DNA Mini Kit (Invitrogen, CA, USA). The viral loads were quantified by using HAdV type-specific real-time qPCR assays. The following cycling conditions were used in the LightCycler 2.0 system (Roche, Penzberg, Germany): an initial denaturing cycle at 94°C for 10 s, followed by 40 cycles of amplification (94°C for 5 s and 60°C for 20 s; the fluorescence was recorded at 60°C). The primers and probe used in this study are as follows. HAdV-55-F: 5’-AGATGAAGAAAGTAAACCGATTT-3’, HAdV-55-R: 5’-CCATCAAGGTCAGTCCAA-3’, HAdV-55-probe 5’-FAM-ATCAGCCAGAACCT CAGCT-BHQ1-3’. The 10-fold serial dilutions of viruses that had been quantitated by the Reed-Muench assay were used as standard samples for standard curve analysis.

### Statistical analysis

Statistical tests were performed using the SPSS 18.0 computer program (IBM, New York, NY, USA, 2009). Data are presented as means ± standard error (SD). All data were analyzed using one-way ANOVA and Dunnett’s test. Significant difference was defined as *p* < 0.05.

## Results

### Adenoviruses isolated from patients with acute respiratory disease

Three outbreaks of acute respiratory disease occurred at military camps in Lasa city/Tibet (January 2016 to March 2016), Shifang city/Sichuan (February 2016 to March 2016) and Kunming city/Yunnan (June 2016 to July 2016) respectively, in China. Overall, more than 300 soldiers were affected in three outbreaks. There were no died cases during the outbreaks. Bioinformatics analysis based on PCR demonstrated that these three outbreaks were all caused by human adenovirus type B55 (HAdV-55). The outbreaks in Tibet and Sichuan continuously occurred. According to the results of epidemiological survey, there are personnel exchanges between military camps in Tibet and Sichuan.

**Fig 1:**
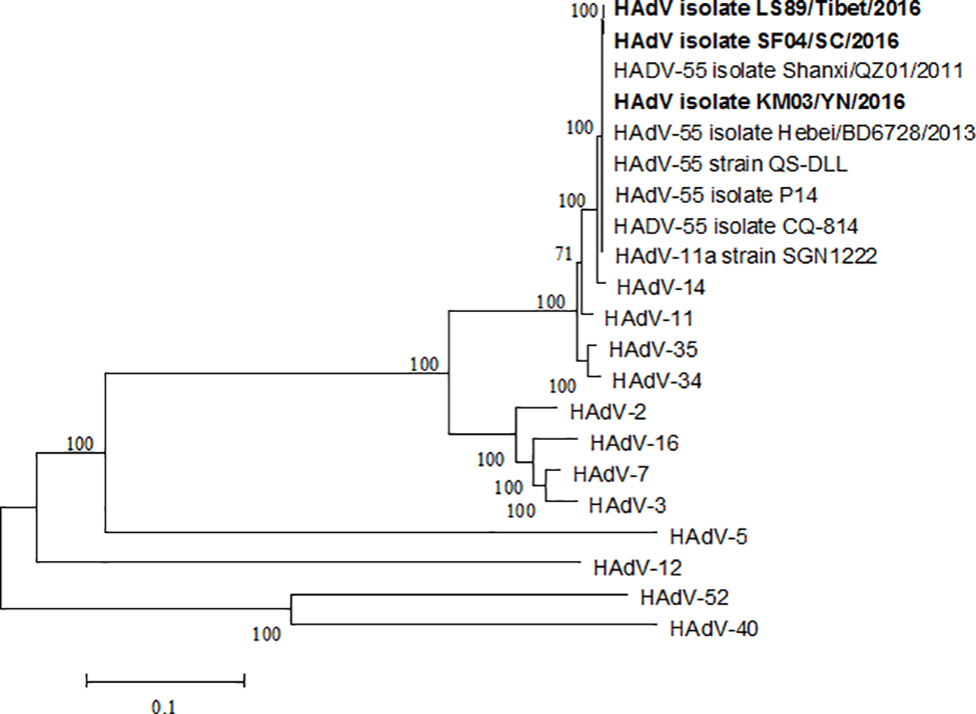
Phylogenetic analysis of isolated HAdV strains. Phylogenetic tree of human adenovirus based on the whole genome sequence was constructed by neighbor-joining method with 1000 bootstrap replicates. The strains isolated in our study are labeled as bold text.

Adenoviruses were isolated from swab samples of patients from Tibet, Sichuan and Yunnan. Three strains of adenoviruses, LS89/Tibet/2016 (GenBank accession no. KY002683), SF04/SC/2016 (GenBank accession no. KY002684) and KM03/YN/2016 (GenBank accession no. KY002685) were obtained and confirmed by wholegenome sequencing. Phylogenetic tree for the whole genome sequences of these virus strains demonstrated that three adenoviruses isolates in this study were all subtype B55 (Fig 1). All previous HAdV-55 strains and our HAdV-55 strains clustered together with previously reported HAdV-14 strains. Our isolates showed a close relationship to some isolates from China (CQ-1657, Shanxi/QZ01/2011, Hebei/BD6729/2013, QS-DLL, CQ814, et al.) and Singapore (SGN1222).

### Genetic characterization of HAdV-55

**Fig 2:**
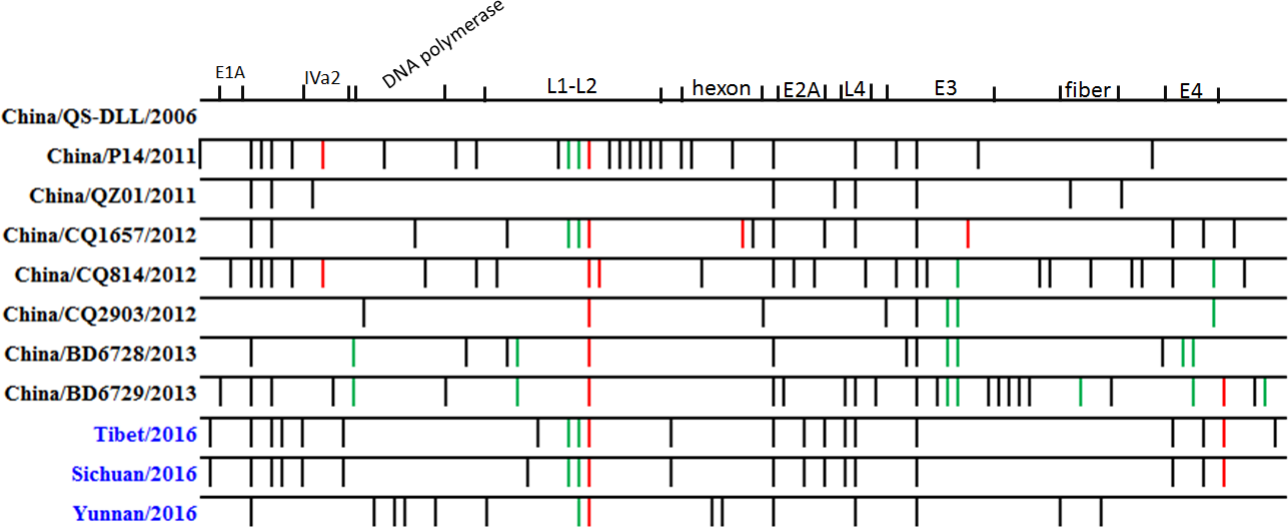
Variation sites based on the China/QS-DLL/2006 strain. The black line represents the mutation, the red line represents base insertion, and the green line represents base deletion.

**Fig 3:**
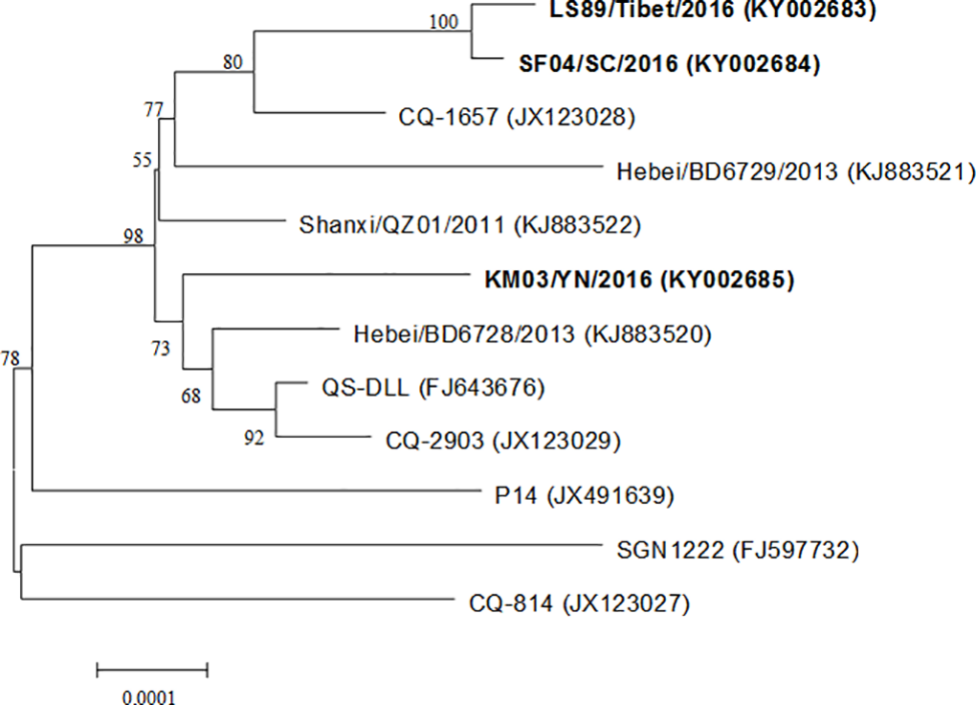
Phylogenetic analysis of HAdV-55 strains. Phylogenetic tree of HAdV-55 strains based on the whole genome sequence was constructed by neighbor-joining method with 1000 bootstrap replicates. The strains isolated in our study are labeled as bold text.

The whole genome sequences of LS89/Tibet/2016, SF04/SC/2016 and KM03/YN/2016 had a homology of 99.8%. The comparisons of variations for all the HAdV-55 whole genome sequences were shown in Fig 2. Mutation sites of these three HAdV-55 strains spread throughout the viral genome, and no multi-gene fragments recombination occurred, which indicates that these HAdV-55 strains were the normal mutations of previous HAdV-55 virus. It is worth to note that LS89/Tibet/2016 and SF04/SC/2016 had a similar mutation pattern, which indicates that the pathogens causing these two outbreaks may be of the same origin. Phylogenetic analysis of HAdV-55 strains also showed that LS89/Tibet/2016 and SF04/SC/2016 were in the same evolutionary branch, while KM03/YN/2016 was different (Fig 3), which further confirmed that LS89/Tibet/2016 and SF04/SC/2016 were of the same origion.

### Phylogenetic analysis of hexon gene and fiber gene

The hexon and fiber protein are the major structural capsid proteins on adenoviruses [13]. Hexon coat proteins are synthesized during late infection and form homo-trimers, and previous studies had indicated that the adenoviruses neutralizing epitopes were likely to be exposed on the surface of the hexon. The fiber protein is one of the major players in adenovirus cell entry. Therefore to further analysis the sequence variation of hexon and fiber gene, phylogenetic tree was constructed using hexon and fiber gene nucleotide sequences. Results showed that three adenoviruses isolates in this study were also typed as B55 subtype (S1 and S2 Figs).

The phylogenetic trees based on hexon and fiber amino acid sequence were consistent with the result of nucleic acid sequences. Moreover, amino acid sequences of hexon and fiber protiens were highly conserved among these HAdV-55 strains. Only one mutation site (aa128, Gly-Cys) was found in hexon protein of KM03/YN/2016 strain compared with other eleven HAdV-55 strains listed in Fig 2, and this mutation site is not located within any of the seven hypervariable regions (HVRs) of hexon gene. The amino acid sequences of fiber protein of the three HAdV-55 isolates in this study were completely consistent with QS-DLL strain. Mutation sites of fiber protein were only found in SGN1222 strain (aa43, Asp-His) and in QZ01 strain (aa207, Thr-Pro).

### Laboratory characteristics of LS89/Tibet/2016, SF04/SC/2016 and KM03/YN/2016

**Fig 4:**
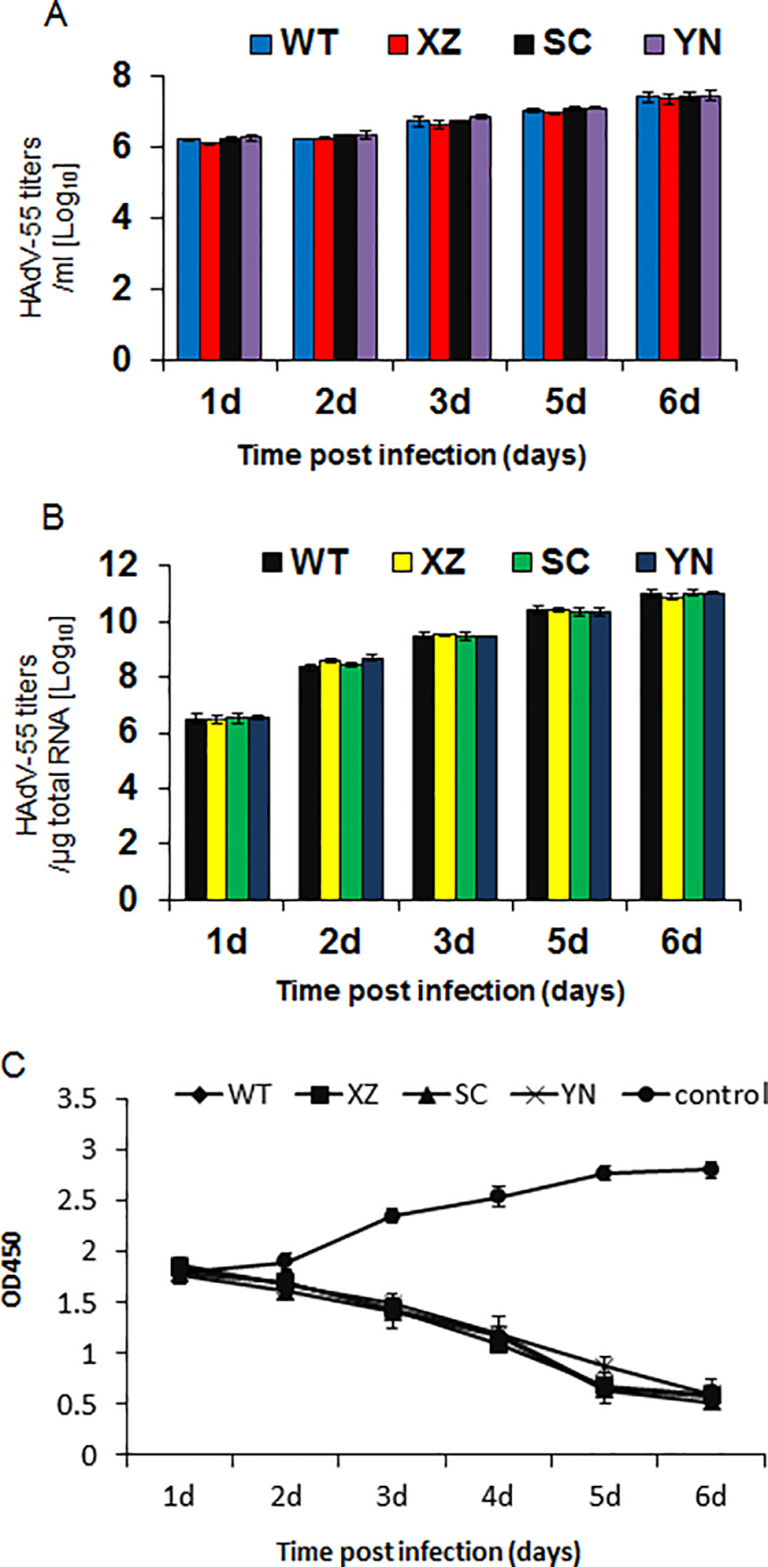
Viral growth kinetics of HAdV-B55 in HEp-2 cells. HEp-2 cells were infected with 100 CCID_50_ HAdV-B55 viruses. The supernatants and cells were separately harvested at 1, 2, 3, 5 and 6 d post-infection. The extracellular viral loads in the supernatants (A) and the intracellular viral loads in the cells (B) were determined by qPCR. The data are shown as the mean 6 standard deviation of three independent experiments. (C) Toxicity of HAdV-B55 in HEp-2 cells. Data were shown the means and standard errors of three replicate assays. WT, XZ, SC and YN present HAdV-55 strains of Y16/SX/2011, LS89/Tibet/2016, SF04/SC/2016 and KM03/YN/2016 respectively.

To further observe the growth kinetics of the three adenoviruses isolates in this study, HEp-2 cells were infected with LS89/Tibet/2016, SF04/SC/2016 and KM03/YN/2016 in the same titers. Supernatants and cells were harvested at 1, 2, 3, 5 and 6 days post-infection, viral DNA levels were determined through real-time qPCR method. As shown in Fig 4A and 4B, these three strains of adenoviruses had similar growth kinetics with a WT HAdV-55 strain in HEp-2 cells. The intracellular levels of adenoviruses increased rapidly by several orders of magnitude after infection. The intracellular and supernatant adenoviruse levels was similar between these isolated HAdV-55 strains and WT HAdV-55 strain after infection by 6 days. Cell viability assay also showed that these three HAdV-55 strains and WT HAdV-55 strain had similar toxicity to HEp-2 cells (Fig 4C).

**Fig 5:**
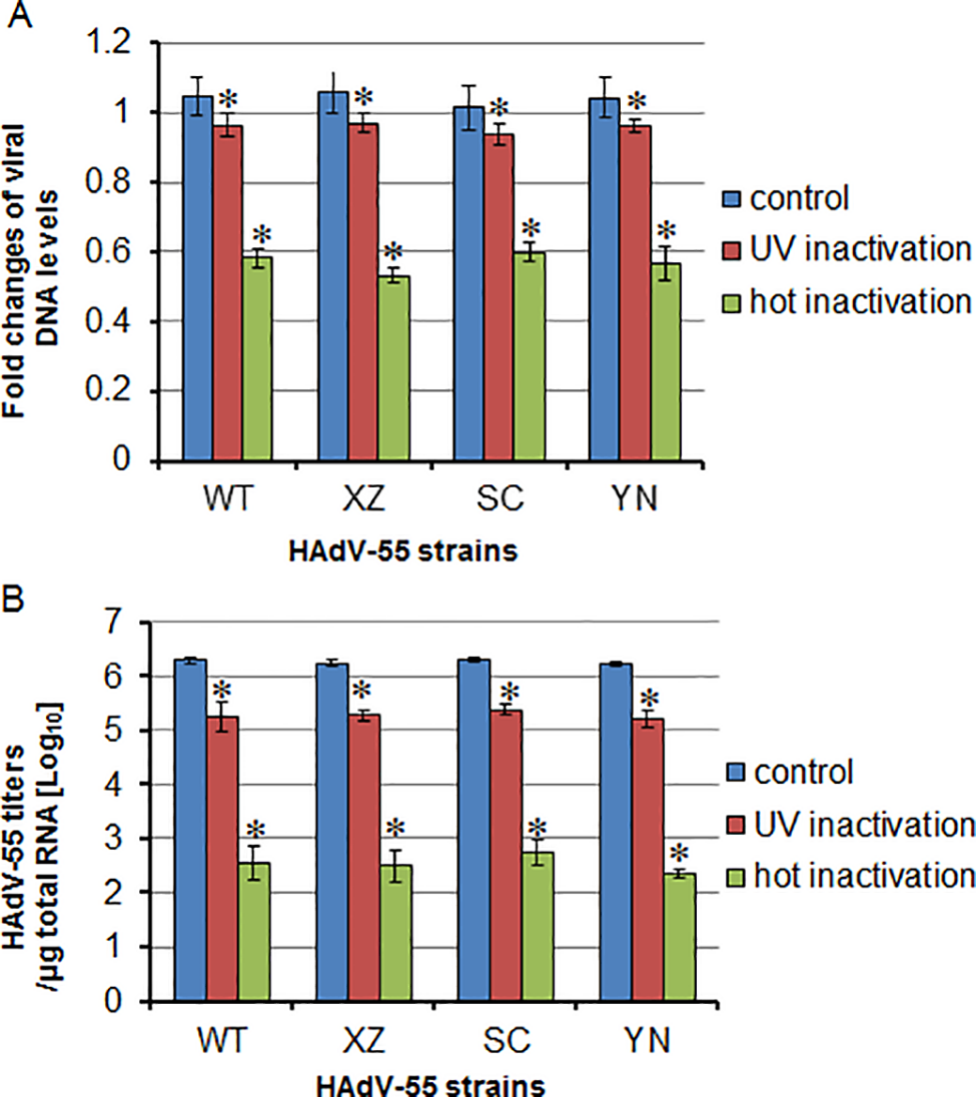
The effect of heat and ultraviolet treatment on the infectivity of HAdV-55. The viruses were incubated for 30 minutes at 56°C or treated for ultraviolet irradiation at a wavelength of 254 nm for 30 minutes. (A) Viral DNA levels after treatment were determined by qPCR. (B) Viral titers in heat- or ultraviolet- treated virus samples were determined by infection on Hep-2 cells. Data were shown the means and standard errors of three replicate assays (* *P* < 0.05, compared with control). WT, XZ, SC and YN present HAdV-55 strains of Y16/SX/2011, LS89/Tibet/2016, SF04/SC/2016 and KM03/YN/2016 respectively.

We further detect the stability of adenoviruses under hot inactivation and ultraviolet irradiation. The viral DNA levels were decreased by 5% and 50% after treated with hot inactivation and ultraviolet irradiation respectively (Fig 5A). HEp-2 cells were infected with these inactive virus and intracellular viral titers were determined after infection by 2 days through real-time qPCR method as shown in Fig 5B. The intracellular levels of virus decreased by almost 10 times after ultraviolet treatment. Interestingly, hot inactivation dramatically depressed the ability of these virus to infect HEp-2 cells. The infection levels of hot inactivated virus decreased by almost 4 orders of magnitude compared with that of control.

## Discussion

Human adenovirus has been recognized as a common cause of acute respiratory disease. Life-threatening adenoviral pneumonia has previously been documented among military trainees, patients with AIDS and transplant recipients. To date, there are at least 69 HAdV genotypes reported, which are classified within seven species (A-G), among which viruses in group B are the common cause of ARD associated outbreaks [1,3].

In the United States, HAdV-3 and HAdV-7 are highly prevalent among children [14]. The prevalence of HAdV has ranged from 0.8% to 11.30% among patients with ARTI in Asia [15,16]. In the past, HAdV-3 and HAdV-7 were also the most prevalent HAdV types in China [4]. Since the first reported HAdV-55 infection in Shanxi province in 2006, severe outbreaks of HAdV-55 associated ARD have been reported sporadically in China [7–10]. HAdV-55 is becoming the leading cause of ARD outbreaks in community, especially in military camps. The clinical manifestation caused by HAdV-55 were closely with other serotypes, such as HAdV-3, HAdV-7 and HAdV-14 [1].

Here, we report three outbreaks caused by HAdV-55 occurred in China in 2016, among which two outbreaks were occurred continuously. One of the outbreaks occurred from January to March 2016 at Lasa city, Tibet, with a sea level above 3658 meters, which is the first report of HAdV-55 outbreak in plateau area. Another outbreak occurred at Shifang city, Sichuan province, from February to March 2016. Epidemiological investigation was carried out immediately after the occurrence of ARD outbreak at Shifang city, and found that three are people coming from the affected areas of the outbreak in Tibet, which is supposed as the cause of the second outbreak. The following whole-genome analyses of these virus strains provides more convincing evidence for this view. The third outbreak occurred from June to July 2016, in Kunming city, Yunnan province. According to the result of epidemiological investigation, the third outbreak was occurred independently from the previous two outbreaks.

To further analyze the source of the pathogens and clarify the mutation characteristics of viral genome sequences, adenoviruses were isolated from swabs of patients from these three outbreaks. The three isolated HAdV strains were named as LS89/Tibet/2016, SF04/SC/2016 and KM03/YN/2016 respectively. The whole genome sequences were obtained and phylogenetic trees were constructed. Through sequencing, homology analysis, and evolutionary tree construction, these three strains have highly similar sequences with previous reported HAdV-55 strains in China. All the three isolated HAdV strains were in the same evolutionary branch with HAdV-55. Result of nucleotide sequences alignment showed that the mutation characteristics of LS89/Tibet/2016 and SF04/SC/2016 were basically consistent, while not the same as KM03/YN/2016. Moreover, the phylogenetic analysis of HAdV-55 strains also showed that LS89/Tibet/2016 and SF04/SC/2016 were in the same evolutionary branch, while KM03/YN/2016 was different. The etiological characteristics were consistent with the results of epidemiological survey, which provides further evidence for the direct relationship between the first two outbreaks. LS89/Tibet/2016 was the first HAdV-55 strain isolated from plateau hypoxia environment. Therefore we particularly concerned about its genetic variation. However, large fragment recombination of the full viral genome was not found, even not so many point mutations in the key proteins such as hexon and fiber. Actually, the real source of LS89/Tibet/2016 was not clear according to the results of epidemiological investigation. The whole-genome analyse results support that the viruses are changed from local epidemic strains by normal mutation.

The infection ability of three isolated HAdV-55 strains to HEp-2 cells was also observed and compared with a WT HAdV-55 strain Y16/SX/2011 which is isolated in Taiyuan city, Shanxi province, China, in 2012. No significant differences were found in the levels of infection among the three HAdV-55 strains and WT HAdV-55 strain. To date, no human adenovirus vaccine is available in China. Heating is one of the ways in which viruses can be made into inactivated vaccines [17,18]. In this study, the isolated HAdV-55 strains could be effectively inactivated by heating at 56°C for 30 minutes, which provides more informations for development of human adenovirus vaccines. In addition, ultraviolet irradiation is also able to reduce the infection ability of HAdV-55, even less effective than hot-inactivation. This supports that UV radiation is to some extent contribute to the prevention of adenovirus, especially in the grass-roots units with relatively limited experimental conditions.

In conclusion, our study demonstrates the characteristics of these HAdV-55 strains from the pathogenic and genetic level. No large gene fragment recombination was found, which indicates that the three strains of HAdV-55 was not belong to a new virus. Heat treatment is an effective way of inactivation of these virus, while UV irradiation could to some extent reduce the infection ability of these viruses. Our study provide more information for genetic evolution and differentiation of HAdV-55, and contribute to prevention and control of HAdV-55 infection in the future.

## Supporting information

S1 Fig Phylogenetic analysis of hexon gene. Phylogenetic tree based on human adenovirus hexon gene (nt 18233–21073, corresponding to the QS-DLL strain) was constructed using neighbor-joining method with 1000 bootstrap replicates. The strains in our study are labeled with the black solid circle.
(PDF)

S2 Fig Phylogenetic analysis of fiber gene. Phylogenetic tree based on human adenovirus fiber gene (nt 30775–31752, corresponding to the QS-DLL strain) was constructed using neighbor-joining method with 1000 bootstrap replicates. The strains in our study are labeled with the black solid circle.
(PDF)

S1 Table Primers used for acquisition of HAdV-B55 whole genome sequence. (PDF)

S2 Table HAdV strains and their GenBank accession numbers used in this study. (PDF)
