# Supplementary figures and images for: Whole-genome analyses of human adenovirus type 55 emerged in Tibet, Sichuan and Yunnan in China, in 2016
Source: PLoS One. 2017 Dec 14;12(12):e0189625. doi: 10.1371/journal.pone.0189625 (PMC5730161; doi:10.1371/journal.pone.0189625)

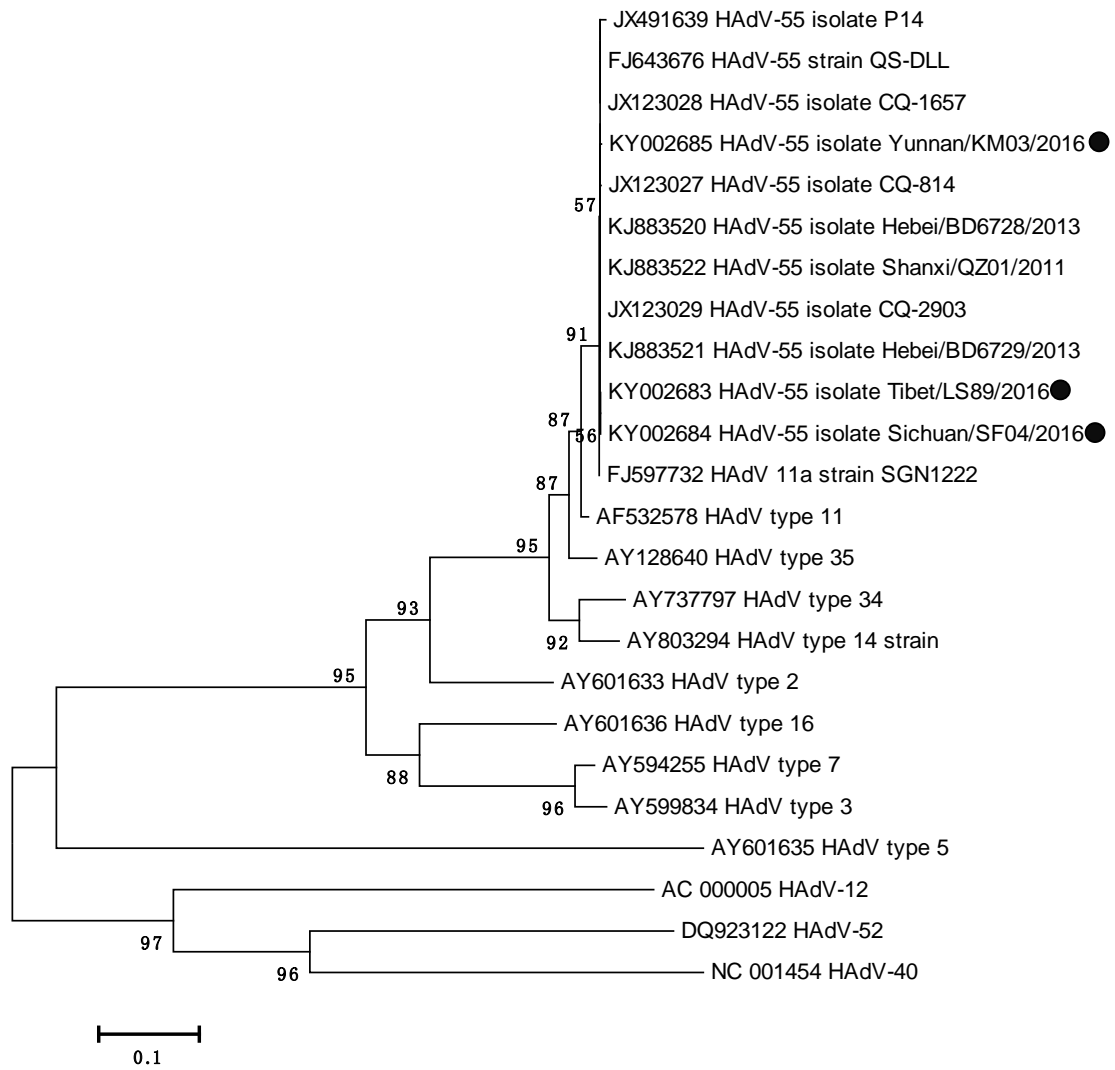

Supplement: S1 Fig — Phylogenetic tree based on human adenovirus hexon gene (nt 18233–21073, corresponding to the QS-DLL strain) was constructed using neighbor-joining method with 1000 bootstrap replicates. The strains in our study are labeled with the black solid circle. (PDF) [file pone.0189625.s001.pdf]

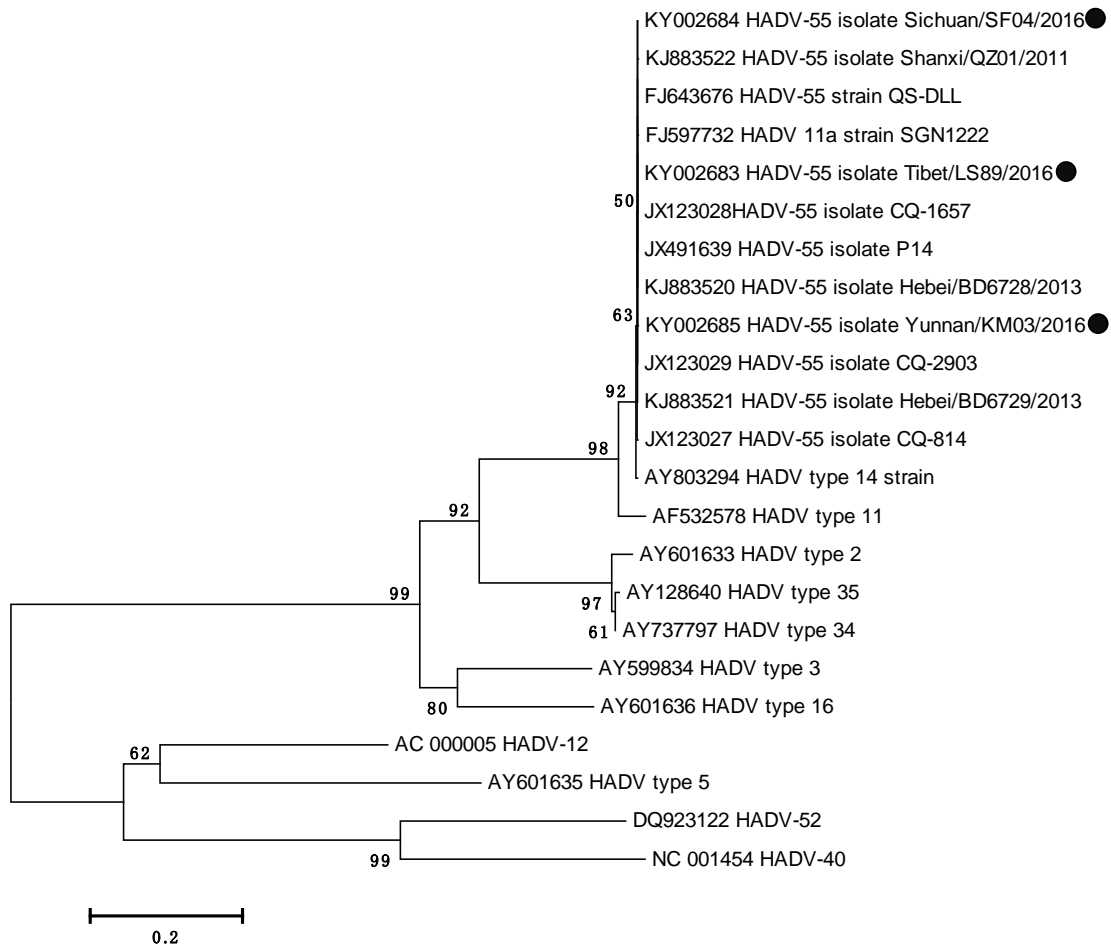

Supplement: S2 Fig — Phylogenetic tree based on human adenovirus fiber gene (nt 30775–31752, corresponding to the QS-DLL strain) was constructed using neighbor-joining method with 1000 bootstrap replicates. The strains in our study are labeled with the black solid circle. (PDF) [file pone.0189625.s002.pdf]
