# Supplementary material for: Whole-genome analyses of human adenovirus type 55 emerged in Tibet, Sichuan and Yunnan in China, in 2016
Source: PLoS One. 2017 Dec 14;12(12):e0189625. doi: 10.1371/journal.pone.0189625 (PMC5730161; doi:10.1371/journal.pone.0189625)
Supplement: S1 Table — (PDF) [file pone.0189625.s003.pdf]

Primers	Sequences (5'to 3')
1-160F	TTTGGGGGTGGAGTGTTTTTGC
1-3340R	AGTGCGGGCAATAACCAAATGAT
2-2973F	ATTTGCGGTGCTTCCGATGAG
2-5860R	GGACCCCAGGTCACGTGAAATAC
3-5690F	GGAGTGCCTCGGTCTTCTTCGT
3-9050R	TCTGGGCGATGTCAGCGAAAT
4-8930F	CATCACCACCTGAGCGAGGTT
4-12320R	ATCAGTCTCAACGCACTCAAAAAGT
5-12120F	CAGATGAGGCCGGACTGGTATAC
5-14910R	GTGACATCTGAGGTGGTGAGCAAT
6-14760F	CGATGTATCTGGAGGAACGGAC
6-18210R	AAGTAACTCAGCGTCGACGCAC
7-18109F	TGCTTTTAATTAAATATGGAGTAGC
7-21516R	AGGAACATGCAGCAGAAAAGT
8-21319F	TTCTAACACCTGCTACCTTTTTGAT
8-24287R	ACAGCGGGGTATGCAAAGTGT
9-24097F	CTTGGAAGAGGTTCCAAAAATCT
9-26876R	TGGATACCTCTGCCTCTGATTAC
10-26636F	TACTTTTGGAACAGTCAGCTCTTAC
10-29862R	TTTCTAAGGTGTTCTGGTGGAGTA
11-29667F	CAACTTCTAGACTGGATCCTTGTG
11-33192R	TCTTCCCTCTCCTCTCCTGCT
12-32904F	AACCTTGTCATAATGGAGTTGCTTC
12-34621R	GTTGCAAGTTAAGCGGATGTGAC
