# Supplementary material for: Whole-genome analyses of human adenovirus type 55 emerged in Tibet, Sichuan and Yunnan in China, in 2016
Source: PLoS One. 2017 Dec 14;12(12):e0189625. doi: 10.1371/journal.pone.0189625 (PMC5730161; doi:10.1371/journal.pone.0189625)
Supplement: S2 Table — (PDF) [file pone.0189625.s004.pdf]

**HAdV strains**	**GenBank accession number**
HAdV-2	AY601633
HAdV-3	AY599834
HAdV-5	AY601635
HAdV-7	AY594255
HAdV-11	AF532578
HAdV-12	AC 000005
HAdV-14	AY803294
HAdV-16	AY601636
HAdV-34	AY737797
HAdV-35	AY128640
HAdV-40	NC 001454
HAdV-52	DQ923122
HAdV-11a strain SGN1222	FJ597732
HAdV-55 strain QS-DLL	FJ643676
HAdV-55 isolate CQ-814	JX123027
HAdV-55 isolate CQ-1657	JX123028
HAdV-55 isolate CQ-2903	JX123029
HAdV-55 isolate P14	JX491639
HAdV-55 isolate Hebei/BD6728/2013	KJ883520
HAdV-55 isolate Shanxi/QZ01/2011	KJ883522
HAdV-55 isolate Hebei/BD6729/2013	KJ883521
HAdV-55 isolate LS89/Tibet/2016	KY002683
HAdV-55 isolate SF04/SC/2016	KY002684
HAdV-55 isolate KM03/YN/2016	KY002685
